# Anatomical Plates

**Published:** 1834-04-01

**Authors:** 


### IV.—(BIS.)

### RECENT ANATOMICAL PLATES.

I. A DEMONSTRATION OF THE NERVES OF THE HUMAN BODY, &c. CONSISTING OF FOUR PARTS. PART IV. THE SPINAL NERVES. By *Joseph Swan*. Price Four Guineas. Longman's, London, 1834.

II. A SERIES OF ANATOMICAL PLATES, IN LITHOGRAPHY, WITH REFERENCES AND PHYSIOLOGICAL COMMENTS, ILLUSTRATING THE STRUCTURE OF THE DIFFERENT PARTS OF THE HUMAN BODY. Edited by *Jones Quain*, M.D. Professor of Anatomy and Physiology in the University of London. Division I. MUSCLES. Fasciculi I. to IX. Price 2s. each Fasciculus.

III. ILLUSTRATIONS OF ALL THE MOST CELEBRATED MEDICAL AND SURGICAL WORKS, COMPRISING A COMPLETE SYSTEM OF MORBID AND DESCRIPTIVE ANATOMY, &c. Fasciculi I. to IX. Price 3*d*. each.

### IV. The Anatomy and Surgery of Inguinal and Femoral Hernia, Illustrated by Plates, coloured from Nature. By *E. W. Tuson,* F.L.S. Assistant-Surgeon to the Middlesex Hospital, &c. Price 2*l*. 2*s*.

Perhaps these are not all the illustrations of anatomy recently published or actually in process of publication in this Country. There may be others which escape our notice, but probably these are brought before the public with the highest recommendations and the fairest promise. When we add to the list the Delineations of Morbid Changes, by Hope, Carswell, and Cruveilhier, we may afford the more retired members of our profession a faint idea of the struggle for profit or for fame, which convulses artists, booksellers, and doctors.

Within these last few years the number of anatomical plates that have been published exceed all rational belief. English, French, Germans, and Americans have successively brought their wares to market. Each and all have deplored the hiatus that remains to be filled up—have pointed out in the strongest terms the wants of the community, and the obvious necessity for a good and cheap series of anatomical engravings—have hinted that they could satisfy those wants and obviate that necessity—yet the last-comer still discovers that his predecessors have not realized the general expectation, and that *he* is exactly the man.

If the general principle of supply and demand is applicable to subjects of this nature, the profession in this Country must experience an insatiable appetite for anatomical plates. System after system appears and is devoured —dropping like the passengers through the crevices and pitfalls of the rotten bridge, in the pleasing allegory of Goldsmith, (we think that it was his,) into the deep waters of oblivion below.

Yet there are some circumstances which lead us to suspect, that the demand is *not* proportioned to the inordinate supply. We have frequently observed the commencement of undertakings of this nature—have gazed with admiration at fasciculus one, two, three—and then the vision has vanished for ever. The surgical world has seen engravings of extremities, to which a trunk has never been appended—trunks, frightful to relate, headless, and with severed limbs.

> Apparent rari nantes in gurgite vasto
> Arma virum, galeæque, et Troia gaza per undas.

The sudden and mysterious disappearance of a predecessor seems to excite no alarm in the breasts of those who follow. Each succeeding speculator mourns over the deficiencies to be supplied—hints that the discriminating public may now expect to be satisfied—promises with confidence and liberality, and descends to the tomb of the Capulets as others did before him.

Seriously, we think that works of this description should not be commenced without due consideration, and a firm resolution to carry them on to their completion. The public have become suspicious, and purchasers, to our own knowledge, have refused to expend their money upon parts till they felt assured of the appearance of the whole. It is a species of gambling, and something akin to a cheat, to entrap unsuspecting persons into laying out their money on imperfect wares. The publisher who sells a single fasciculus of a work which he says will be completed, does morally guarantee the completion to the buyer. If he breaks his word, there is probably little immediate redress, but retributive justice overtakes the trade in the suspicious unwillingness of the public to purchase.

We fear that another unpleasant reflection presents itself to the mind of the most kindly critic. He cannot but question the justice of the idea, that anatomical plates of the description offered by many booksellers, are really acquisitions to the youth of the profession.—"Cheap and nasty," as many of them have been, we think they have proved dear in reality to the buyers. The best judges have come to the conclusion that if any more anatomical engravings are wanted, they should be of the very highest order. We have had too much from the auction mart already. It is true that the expense would be considerable, frightful—that the present sale would be doubtful and inadequate—that no man in trade, perhaps no individual whatever, would be justified in incurring the outlay of capital or the expenditure of labour. We entertain no doubt that they would pay at last, but we could not dare to define the period.

The College of Surgeons might undertake this national work—might consent to sacrifice a portion of its funds, in order to render it accessible to those of moderate means. The College possesses facilities which no individual can obtain—might do the most at the least expense—might benefit the profession without material loss to itself, perhaps without any ultimate loss whatever. This is one of many boons which the College might confer upon its members—which some contend that it should. We hope to see the day when the College will be foremost in works of utility, and when its power will be exerted to the uttermost in vindicating the honour of the profession, as a learned and accomplished body, in stretching its helping hand to needy merit, and in ardently promoting the interests of science.

At the head of the works before us is that of Mr. Swan. It has left all competitors at an immeasurable distance.

> Unde nil majus generatur ipso,
> Nec viget quicquam simile aut secundum.

If a national system of anatomical plates were constructed, these of Mr. Swan should form a component part of it. If the other portions of the body were delineated with the same exactness and the same effect, we might challenge the world to produce their superiors.

The part before us, the fourth and last, is devoted to the exhibition of the anatomy of the spinal nerves. It consists of nine copper-plate engravings, and as many illustrative outlines. Of the execution we need say little. It equals that of the preceding parts; and to say this, is to express that it excels all other anatomical engravings that have ever been presented to the world, in this country or in any other. Criticism, in the instance of these drawings and engravings, is surprized to find herself metamorphosed to the language of encomium. Yet the modesty of Mr. Swan deems it necessary to apologize thus for the artist.

"In the prosecution of this work, every endeavour has been used for representing clearly and accurately the complicated preparations on which it has been entirely founded. If instruction is the principal object of anatomical plates, and not the gratification of the imagination, distinctness must be the leading feature; and, therefore, the rules of the artist must, in some measure, be infringed upon, as it is often impossible to give all the minute parts clearly in detail, and preserve the proportionate degrees of light and shade necessary for the perfection of the whole."

With the concluding portion of this unaffected passage we cordially agree.

"So long as the principal objects are intelligibly delineated, plates will convey such information respecting the precise situation of the most minute and intricate parts as cannot be derived from words, however carefully and judiciously these may have been selected and arranged."

There can be no question of the utility of good plates, even to him who dissects with zeal. There can be little doubt of the danger of bad ones.

Did we feel inclined to point out individual excellencies, we would turn to the delineations of the nerves of the neck, the shoulder, the arm, and the foot. In the instance of the latter and the hand, their anatomy is displayed with a clearness which robs it of all difficulty or confusion.

Mr. Swan's noble work is now brought to a conclusion. He deserves the warmest thanks of the profession—the kindest encouragement and patronage from the College. We trust he will have both. In these splendid plates there is much to praise—the patience and the toil of the dissector, the skill of the engraver. Censure's occupation's gone, and probably there is little even to amend. But we think that there are some omissions—that more plates might be added—that some minute points might be more completely illustrated. The distribution of the suboccipital and second and third cervical nerves might be farther shewn—the sacral nerves might perhaps be displayed with more exactness. In short, the only fault that can possibly be found with Mr. Swan is one of a flattering description—we have not quite enough of him.

Nine Fasciculi of Mr. Quain's plates are before us. The responsible situation which this gentleman fills, with honour to himself and advantage to his class, is a guarantee for their completion, were no other present. But the character and the popularity of the work are amply sufficient to ensure its continuance.

The plates are lithographed, of folio size, that is, of nearly natural dimensions, and two are contained in each fasciculus. The nine now lying before us display the muscles of the face, head, neck, of the larynx and the pharynx, and of the upper extremity. The number of fasciculi will be about one hundred and twenty-five, or say one hundred and thirty; and, as each is to cost two shillings, the expense of the whole will amount to about thirteen pounds. Now this, for lithography, is no inconsiderable sum, and the purchaser has a right to expect that the execution shall be of a high order. We are sorry to say that it is not—we are compelled to animadvert on the smudgy indistinctness of many of the plates, and the very indifferent appearance of the majority. It must be remembered that all, or almost all, are copies—that lithography is cheap—that the plates of Cloquet and of Knox are already in the market. We repeat that these plates are not what they should be. As the names of the artists are brought prominently forward by Mr. Quain, in his prospectus, our readers may be gratified at learning that the drawings are by Mr. W. Fairland, the printing by Hulmandel. We think it would have been more charitable in Mr. Quain to have spared the modest blushes of these gentlemen. We are induced to make these animadversions, because the work might be much improved, and because, for its price, it should be so. On a future occasion, we shall take an opportunity of noticing the amendment, which we do not doubt will be effected.

We differ from Mr. Quain in one important particular. With the referential description of the plates, he has mixed up discourses de omnibus rebus, et quibusdam aliis. Physiology, pathology, dislocations, &c. &c. are treated of. We do not hesitate to term this a *bore*, a positive nuisance. God knows there are sciolists enough now-a-days, without this unceasing attempt to manufacture more. Turn to what book we will the title-page takes us in. If a work of anatomy appear, it is sure to be inflated to a most unnatural bulk with odds and ends of pathology and surgery:—

Like some patch'd dog-hole, eked with ends of wall.

Let our medical cobblers stick to their lasts—let them, for Heaven's sake, do what they profess, and do no more. We do not look to anatomical plates for a treatise upon dislocations. There are able works enough on that subject, to say nothing of the manuals and sub-manuals which are pelted at the brains of students from every shop-window.

Enough o' this, Hal, an you love me.

Of the ILLUSTRATIONS OF ALL THE MOST CELEBRATED MEDICAL AND SURGICAL WORKS, we will only say, that we think the speculation can never succeed. We suspect that more money has been lost in second and third-rate undertakings, than would probably have rendered the success of a first-rate work secure.

Mr. Tuson's mode of shewing the anatomy of inguinal and femoral hernia by layers is ingenious. He has been anticipated by Mr. Bloxham, whose book was noticed in a late Number of this Journal. Can the anatomy of hernia be learnt upon paper?

It may seem that some of our remarks have been ill-natured. We will say, once for all, that there is no Journal, daily, weekly, or quarterly—medical or general, in which considerations of a private description have less influence than in this. We state it openly and honestly. Such is the condition of medical literature, that the pruning knife is become too necessary. The critic that applies it must expect some odium; but, if he possesses any love for his profession, he has no alternative. None can be more ready than ourselves to bestow deserved eulogy—none *do* bestow it with more warmth. It is with pain we censure, and we censure to improve. With the candid and the liberal, let this be our apology.

